# PRODH Regulates Tamoxifen Resistance through Ferroptosis in Breast Cancer Cells

**DOI:** 10.3390/genes15101316

**Published:** 2024-10-14

**Authors:** Ping Zhang, Na Qian, Haigen Lai, Shu Chen, Kuaiying Wu, Xiaofeng Luo, Bo Lei, Mengqi Liu, Jiajun Cui

**Affiliations:** The Department of Biochemistry, Medicine School, Yichun University, Yichun 336000, Chinaqn1033217157@outlook.com (N.Q.); wukuaiying@163.com (K.W.); xiaofeng_luo619@outlook.com (X.L.); bl0120@163.com (B.L.); meng3341083882@outlook.com (M.L.)

**Keywords:** proline, PRODH, tamoxifen resistance, ferroptosis

## Abstract

Background: Estrogen receptor-positive breast cancer accounts for around 70% of all cases. Tamoxifen, an anti-estrogenic inhibitor, is the primary drug used for this type of breast cancer treatment. However, tamoxifen resistance is a major challenge in clinics. Metabolic reprogramming, an emerging hallmark of cancer, plays a key role in cancer initiation, progression, and therapy resistance. The metabolism of non-essential amino acids such as serine, proline, and glutamine is involved in tumor metabolism reprogramming. Although the association of glutamine metabolism with tamoxifen resistance has been well established, the role of proline metabolism and its critical enzyme PRODH is unknown. Objective: The aim of this study is to explore the role and mechanism of PRODH in tamoxifen resistance in breast cancer cells. Methods: PRODH and GPX4 expressions in tamoxifen-resistant cells were detected using real-time PCR and Western blot analysis. The breast cells’ response to tamoxifen was measured using MTT assays. Trans-well assays were used to detect cell migration and invasion. A Xenograft tumor assay was used to detect the role of PRODH in tumor growth. Reactive oxygen species were measured using flow cytometry. Results: PRODH expression is reduced in tamoxifen-resistant cells, and its overexpression enhances tamoxifen response in vitro and in vivo. Conversely, PRODH knockdown confers tamoxifen resistance in tamoxifen-sensitive cells. Mechanistic studies show that ferroptosis is inhibited in tamoxifen-resistant cells and overexpression of PRODH restores the ferroptosis in tamoxifen-resistant cells. Moreover, Ferrostatin-1 (Fer-1), the ferroptosis inhibitor, reversed the effect of PRODH on tamoxifen resistance. Conclusions: These findings suggest that PRODH regulates tamoxifen resistance by regulating ferroptosis in tamoxifen-resistant cells.

## 1. Introduction

Breast cancer is the most common malignancy and the second leading cause of death in women. The majority of breast cancer is estrogen-receptor positive [1,2,3]. Tamoxifen, an anti-estrogenic inhibitor, is commonly used to treat this type of breast cancer. Unfortunately, the occurrence of acquired resistance in many patients is inevitable [4,5]. Thus, the resistance to tamoxifen treatment is a major challenge in breast cancer. Given the complex biology of the ER and the acquisition of tamoxifen resistance, the mechanisms under tamoxifen resistance in breast cancer are still not fully understood [6,7].

Metabolic reprogramming is critical for fueling tumor growth. The metabolism of cancer cells exhibits a distinct characteristic compared to normal cells. Increasing studies show that cancer cells rely heavily on the use of amino acid catabolism to maintain their proliferation. This gives cancer cells advantages such as rapid proliferation, anti-apoptosis, and inhibition of anti-cancer immune effects [8,9,10]. The metabolism of non-essential amino acids (NEAAs), namely serine, proline, and glutamine, is involved in tumor metabolism reprogramming [11,12]. Proline is substantially increased in cancer cells and its metabolism is essential for cancer cell proliferation, survival, and metastatic spread [13,14].

Proline dehydrogenase (PRODH), also known as proline oxidase (POX), is tightly bound to the mitochondrial inner membrane. PRODH is the enzyme responsible for the first step of proline catabolism, catalyzing proline oxidation and converting it to ∆ 1-pyrrolidine-5-carboxylate (P5C) in the mitochondria. During this reaction, generated electrons are either transported to the respiratory chain to produce ATP or directly reduce oxygen to produce reactive oxygen species (ROS). It has been shown that PRODH-dependent ATP production promotes tumor cell survival in energy stress, while PRODH-dependent ROS production induces cancer cell apoptosis and functions as a tumor suppressor [15,16,17,18]. Thus, PRODH may play a dual role in cancers. Since PRODH was firstly discovered as a p53-induced gene, accumulating evidence shows that PRODH is down-regulated in many types of human tumors such as renal and liver cancer [16,19,20]. However, in other types of cancers such as lung and pancreas cancer, PRODH is up-regulated and functions as a tumor promoter [21,22]. In breast cancer, PRODH promotes apoptosis in breast cancer cells and is correlated with a better prognosis [23,24].

Although the role of glutamine metabolism in tamoxifen resistance has been identified, the effect of proline metabolism and PRODH in tamoxifen resistance is unknown. Here, our data suggest that PRODH exhibits a low level in tamoxifen-resistant cells, and PRODH overexpression restores the response to tamoxifen. More importantly, we found that ferroptosis induced by PRODH could potentially overcome tamoxifen resistance in the treatment of breast cancer.

## 2. Materials and Methods

### 2.1. Cell Culture

Cell lines T47D and MCF-7 were obtained from the Cell Bank of Chinese Academy of Sciences. STR profiling was used to authenticate cell lines [25]. Cells were maintained in Dulbecco’s modified Eagle’s medium (DMEM) with 10% FBS. Tamoxifen-resistant cells T47D/TamR and MCF-7/TamR were acquired by maintaining cells in 0.1–1 μM tamoxifen over 12 months, as described previously [25]. Prior to 4-Hydroxytamoxifen (TAM) treatment, cells were maintained in phenol red-free DMEM with charcoal-stripped FBS.

### 2.2. Lentivirus Package and Transfection

PRODH cDNA was inserted into pCDH-CMV, this construct was then used to produce lentivirus in 293T cells [25]. PRODH-specific shRNA were purchased from Genetech and were cloned into hU6-MCS-CMV-puromycin for lentivirus production. For lentivirus packaging, PRODH overexpression or shRNA-containing plasmids, along with lentiviral packaging plasmids psPAX2 and pMD2.G, were co-transfected into 293T cells using lipofectamine 3000 transfection reagent (Invitrogen, Carlsbad, CA, USA). The lentivirus supernatant was collected 36 h later. Breast cancer cells were then infected with lentivirus particles with 10 μg/mL polybrene (Solarbio, Beijing, China). Stable expression cell lines were generated using 2 μg/mL puromycin (Sigma, St. Louis, MO, USA).

### 2.3. Real-Time PCR

RNA was extracted using an Rneasy Mini kit (Qiagen, Hilden, Germany) and reverse transcription was performed using SuperScript^®^ III Reverse Transcriptase (Invitrogen). Primers for PRODH were as follows: 5′-GGATGCCTATGACAATG-3′ and 5′-CCTTGGCGTTGTGCTTC-3′ [26]. Primers for GPX4 were as follows: 5′-GAGGCAAGACCGAAGTAAACTAC-3′ and 5′-CCGAACTGGTTACACGGGAA-3′. The expression of genes of interest was performed using the ABI Prism 7700 Detection System (Applied Biosystems, Foster City, CA, USA).

### 2.4. Western Blot

Cells were collected and lysed in RIPA buffer (Beyotime, China) for 30 min on ice. Samples were mixed with SDS-loading buffer and boiled for 10 min. After centrifugation, proteins were separated by SDS-PAGE and transferred to PDVF membrane. The membrane was incubated with antibodies as indicated. The ECLplus Western Blot Detection System (Beyotime, China) was employed to visualize protein bands. The antibodies against PRODH, glutathione peroxidase 4(GPX4), and Actin were obtained from Abcam (Cambridge, UK), Proteintech (Wuhan, China), and Beyotime (Shanghai, China).

### 2.5. MTT Assay

MTT (Sigma-Aldrich, St. Louis, MO, USA) was used for cell proliferation. A total of 5000 cells were plated in each well of a 96-well plate and treated with tamoxifen or control. Five days later, MTT was added to the medium and incubated for 4 h. The medium was then discarded and replaced with 0.2 mL DMSO. After incubation for 30 min at room temperature, cell viability was assessed by measuring the absorbance at a wavelength of 570 nm using a microplate reader.

### 2.6. Soft Agar Assay

DMEM complete medium and 1.2% agar were mixed and plated in a 6-well plate as the bottom agar layer. A total of 5000 cells in DMEM were mixed with 0.6% low-melting agarose and then plated on the bottom agar layer. Complete medium was added to the top of the soft agar. Every 3 days, medium was replaced with fresh medium. Cells were incubated for around 2 weeks. The resulting colonies were then observed and counted under a microscope.

### 2.7. Proline Measurement

A Proline Assay kit (Arigo Bio laboratories, Hsinchu City, Taiwan) was used for proline content measurement. Briefly, cells were harvested and washed with PBS. After centrifugation of the assay buffer, pre 5 × 10^6^ cells were added into the tube and then samples were sonicated. To extract proline, the samples were heated in a boiling water bath for 10 min. After centrifugation, the supernatants were moved into new tubes and stood on ice. Standards and samples were incubated with reaction solution and reaction dye at 90 °C in the dark for 20 min. A synergy spectrophotometer was used to measure the OD of the conversion dye at 520 nm.

### 2.8. Intracellular GSH Measurement

Glutathione (GSH) levels were detected using a Glutathione Assay Kit (Jiancheng Biotechnology Institute, Nanjing, China). 1 × 10^4^ MCF-7/TAMR cells expressing PRODH were plated in a 24-well plate and then treated with tamoxifen and ferrostatin-1. Cells were then harvested and homogenized in PBS. After centrifugation, the supernatants were used to measure GSH levels. A microplate reader (Biolab, GA, USA) was used to detect the absorbance of 450 nm.

### 2.9. Intracellular ROS Assay

A Reactive Oxygen Species Assay Kit (UEland, Suzhou, China) was employed to measure ROS levels. Briefly, cell suspension was dyed with DCFH-DA staining solution for 1 h. After washing twice using PBS, flow cytometry assays were performed to detect ROS levels.

### 2.10. Ethics Statement and Mouse Care

This protocol was approved by the Ethical Committee of Yichun University Under approval number IACUC-2019035. The Animal Care and Use Protocol complied with the ARRIVE guidelines, the U.K. Animals (Scientific Procedures) Act 1986, and associated guidelines, as well as the EU Directive 2010/63/EU for animal experiments. Balb/c nude mice were maintained in a pathogen-free environment. Mice were monitored daily for health issues such as weight loss, loss of mobility, etc. Health issues and tumor overgrowth (the long diameter > 12 mm) were set as the humane endpoint. Once the endpoint was reached, mice were immediately euthanized by gradually increasing the concentration of CO_2_ in the anesthesia chamber. All efforts were made to minimize animal discomfort.

### 2.11. Animal Experiments

Female nude mice were injected with 1 × 10^6^ MCF-7/TamR cells expressing PRODH or empty vector. When tumors reached a size of about 80 mm^3^, mice were randomized into groups to receive tamoxifen pellets and/or ferrostatin-1 (fer-1) (Aladdin, Shanghai, China) as indicated. A digital caliper was used to measure tumor progression twice a week. After 7 weeks, mice were euthanized and excised tumors were weighed.

### 2.12. Statistical Analysis

Statistical analysis and plotting were conducted using Graphpad Prism7.0. A one way ANOVA and Student’s *t*-test were used for group comparison. All the experiments were performed at least in triplicate. Data were shown as mean ± SD. *p* < 0.05 was established as the threshold for statistical significance.

## 3. Results

### 3.1. PRODH Expression Is Reduced in Tamoxifen-Resistant Cells

**Figure 1:**
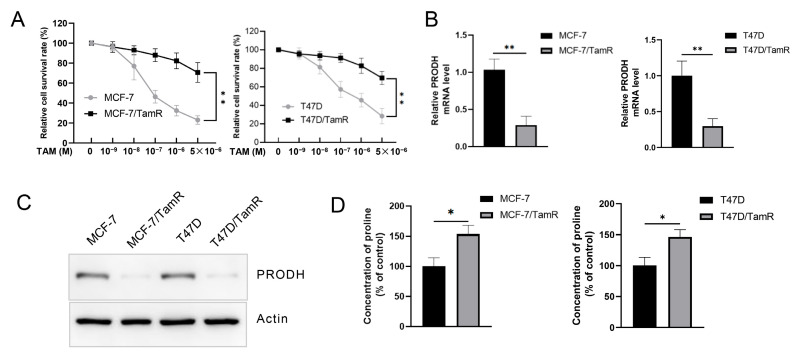
PRODH is down-regulated in tamoxifen-resistant cells. (**A**) T47D/TamR and MCF-7/TamR were established and the response to tamoxifen was measured using an MTT assay. (**B**,**C**) PRODH expression in tamoxifen-resistant cells by real-time PCR (**B**) and Western blot (**C**). (**D**) Proline concentration in T47D/TamR and MCF-7/TamR cells was detected. * *p* < 0.05; ** *p* < 0.01.

Recent studies suggest the key role of PRODH in breast cancer. To study the effect of PRODH on tamoxifen resistance, we developed tamoxifen-resistant MCF-7/TamR and T47D/TamR cells (Figure 1A). Then, PRODH expression was detected in these cells. As a result, PRODH expression decreased significantly at mRNA and protein levels in resistant cells compared to their parent cells (Figure 1B,C). PRODH is a key enzyme to degrade proline. Thus, we measured the proline concentration in these resistant cells. As expected, the increase in proline concentration was observed in these two tamoxifen-resistant cell lines (Figure 1D).

### 3.2. PRODH Regulates Tamoxifen Resistance in Breast Cancer Cells

**Figure 2:**
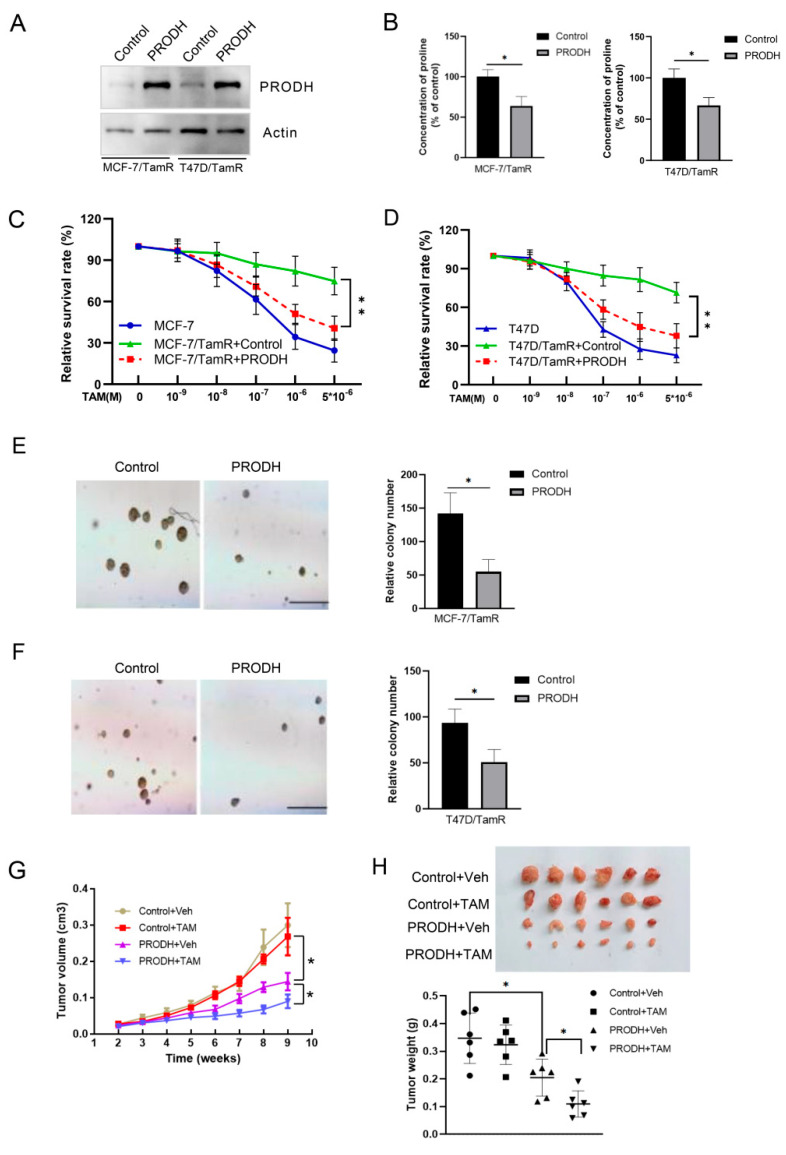
PRODH modulates tamoxifen response in breast cancer cells. (**A**) Tamoxifen-resistant cells were infected with PRODH containing lentivirus, and the expressions of PRODH were detected using Western blotting. (**B**) Proline concentrations were measured in cells as indicated in (**A**). (**C**,**D**) T47-D/TamR and MCF-7/TamR cells expressing PRODH were treated by TAM for 5 days. MTT assays were conducted to measure cell proliferation. (**E**,**F**), Anchor-independent growth was detected using soft agar assay in T474D/TamR MCF-7/TamR cells expressing PRODH. The scale bar indicates 0.1 mm. (**G**) The impact of PRODH on tumor growth under tamoxifen treatment. MCF-7/TamR cells expressing PRODH were injected into nude mice and were randomly assigned to 4 groups as indicated and treated with and without tamoxifen. (**H**), Upon reaching the endpoint, mice were euthanized, and tumors were weighed. * *p* < 0.05; ** *p* < 0.01.

To study the effect of PRODH in tamoxifen response, we infected tamoxifen-resistant cells with lentivirus expressing PRODH or control vector (Figure 2A). As expected, we found that re-expression of PRODH decreased proline concentration in T47D/TamR and MCF-7/TamR cells (Figure 2B). We also discovered that re-expression of PRODH sensitized resistant cells to tamoxifen treatment (Figure 2C,D). In addition, soft agar assays revealed that PRODH significantly reduced anchor-independent growth of resistant cells during tamoxifen treatment (Figure 2E,F).

To study the impact of PRODH on tamoxifen response in vivo, xenografts of PRODH overexpression MCF-7/TamR cells were performed. As expected, tamoxifen treatment produced no effect on tumor growth derived from tamoxifen-resistant cells. However, tamoxifen significantly amplified the growth-inhibitory effects of PRODH overexpression (Figure 2G,H). Together, these results identify the key role of PRODH expression in tamoxifen resistance.

### 3.3. Knockdown of PRODH Renders Cells Resistant to Tamoxifen

**Figure 3:**
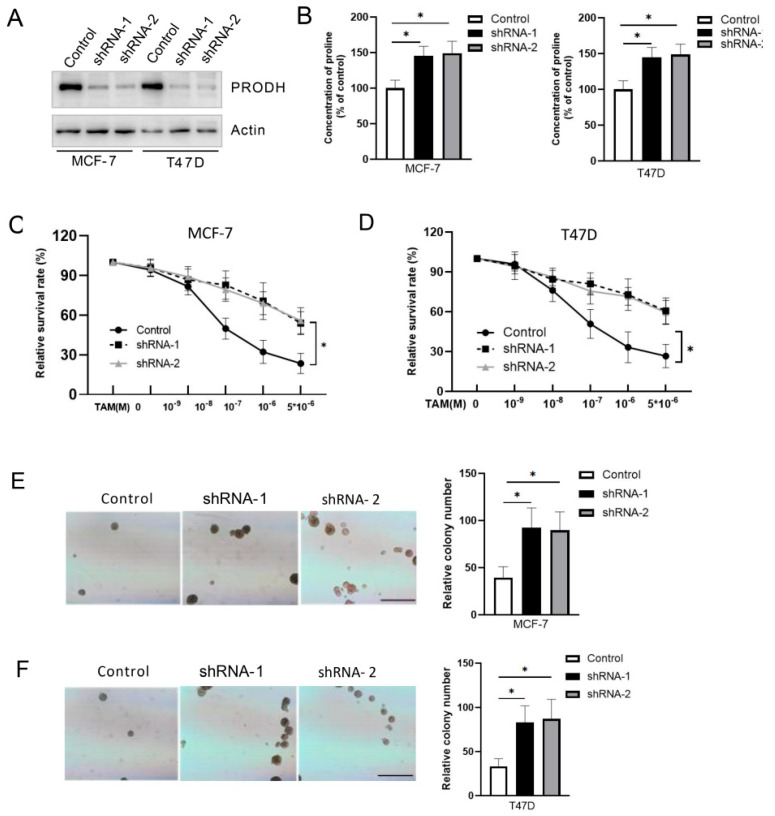
Knockdown of PRODH renders cells resistant to tamoxifen. (**A**) PRODH expression in T47D and MCF-7 cells expressing PRODH shRNA-1 and -2 was detected by Western blot. (**B**) The proline concentrations were measured in cells as indicated in (**A**). (**C**,**D**) MCF-7 and T47-D cells were treated by PRODH shRNA-1 and -2 containing lentiviruses and treated with TAM for 5 days. MTT assays were conducted to measure cell proliferation. (**E**,**F**) Anchor-independent growth was detected by soft agar assay in T474D and MCF-7 cells. The scale bar indicates 0.1 mm. The scale bar represents 100 nm. * *p* < 0.05.

To confirm the role of PRODH in the tamoxifen response in breast cancer cells, MCF-7 and T47D cells were infected with lentivirus containing PRODH-specific shRNA. As result, shRNA treatments significantly reduced PRODH protein levels in these cells (Figure 3A). Then, we measured proline concentrations and found that PRODH knockdown increased proline concentrations in T47D and MCF-7 cells (Figure 3B). More importantly, the dose–response curves show that tamoxifen had much less effect on shRNA-treated cell survival (Figure 3C,D). We also found that PRODH knockdown increased anchor-independent growth of T47D and MCF-7 cells under tamoxifen treatment (Figure 3E,F). These results suggest that PRODH knockdown is associated with tamoxifen resistance in breast cancer cells.

### 3.4. PRODH Reduces the Migration and Invasion of Tamoxifen-Resistant Cells

**Figure 4:**
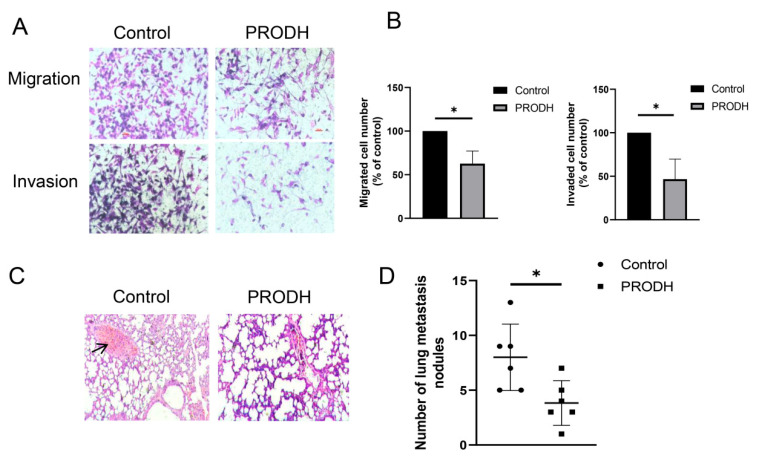
PRODH suppresses the migration and invasion of tamoxifen-resistant cells. (**A**) Cell migration assay in PRODH overexpression MCF-7/TamR or control cells. (**B**) Cell invasion assay in PRODH overexpression MCF-7/TamR or control cells. (**C**,**D**) The effect of PRODH on lung metastasis of tamoxifen-resistant-cell-derived tumors. PRODH overexpression MCF-7/TamR cells in nude mice and lung metastasis were measured by H&E staining (**C**), and the nodules were counted under a microscope (**D**). The arrows indicate metastatic nodules. * *p* < 0.05.

A recent study showed that higher cell migration and invasion are linked to acquired tamoxifen resistance [27]. Thus, we decided to investigate the role of PRODH on cell migration and invasion in tamoxifen-resistant cells (Figure 4A,B). As a result, PRODH significantly reduced cell migration and invasion in MCF-7/TamR cells (Figure 4A,B). Breast tumors often exhibit higher proportions of lung metastasis. Next, we injected PRODH overexpression MCF-7/TamR cells into nude mice to detect the effect of PRODH on the metastasis of tamoxifen-resistant cells in vivo. The number of nodules in the lungs was clearly decreased in the PRODH overexpression group (Figure 4C,D).

### 3.5. PRODH Induces Ferroptosis in Tamoxifen-Resistant Cells

**Figure 5:**
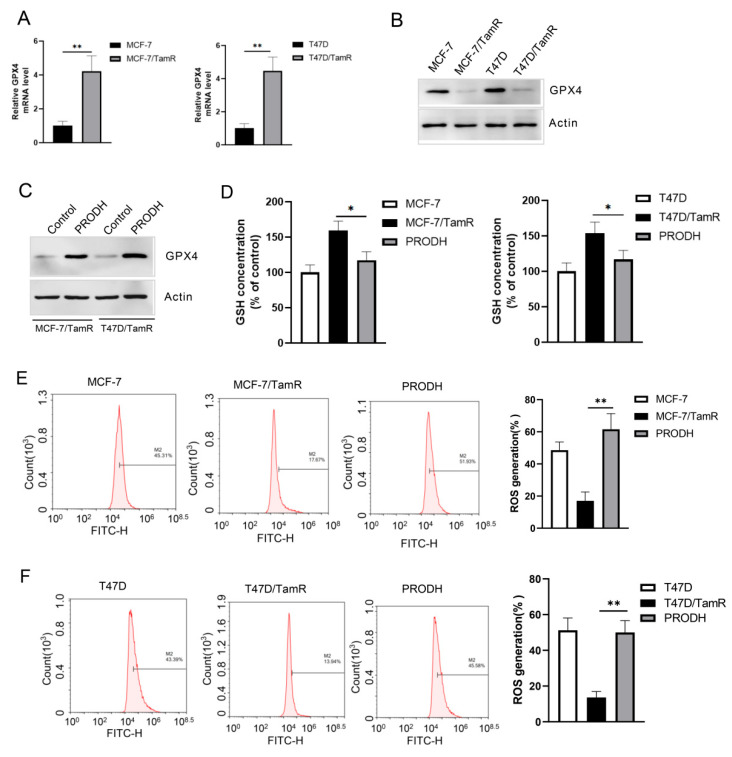
PRODH induces ferroptosis in tamoxifen-resistant cells. (**A**) Relative GPX4 mRNA levels were detected using real-time PCR in MCF-7/TamR and T47D/TamR cells. (**B**) GPX4 expression was measured using Western blot in tamoxifen-resistant cells. (**C**) MCF-7/TamR and T47D/TamR cells were infected by PRODH expressing lentivirus and the expressions of GPX4 were detected by Western blot. (**D**) MCF-7/TamR and T47D/TamR cells were infected with lentivirus expressing PRODH and the GSH concentration was measured. (**E**,**F**) MCF-7/TamR and T47D/TamR cells were infected with lentivirus expressing PRODH and the ROS levels were detected using flow cytometry. * *p* < 0.05; ** *p* < 0.01.

Ferroptosis is a key regulator in therapy resistance of cancer treatments. Therefore, we decided to investigate the alteration of ferroptosis in tamoxifen-resistant cells and its possible link with PRODH. To do this, we first detected the expression of the ferroptosis marker genes using real-time PCR and Western blotting. GPX4 exhibited higher levels of both mRNA and protein in resistant cells compared to their parent cells (Figure 5A,B). Following this, the effect of PRODH on GPX4 expressions was investigated using Western blot analysis. As shown in Figure 5C, re-expression of PRODH led to a significant decrease in GPX4 expression.

Ferroptosis is characterized by a high level of intracellular ROS through the depletion of GSH. Therefore, we conducted experiments to measure ROS and GSH levels in tamoxifen-resistant cells. As a result, an increase in GSH concentration and a decrease in ROS production were observed in MCF-7/TamR and T47D/TamR cells compared to their parent cells. Overexpression of PRODH diminished these alterations in resistant cells (Figure 5D–F). These findings suggest that tamoxifen-resistant cells exhibit reduced ferroptosis, and re-expression of PRODH reverses the alteration of ferroptosis in tamoxifen-resistant cells.

### 3.6. PRODH Regulates Tamoxifen Resistance through Ferroptosis in Breast Cancer Cells

**Figure 6:**
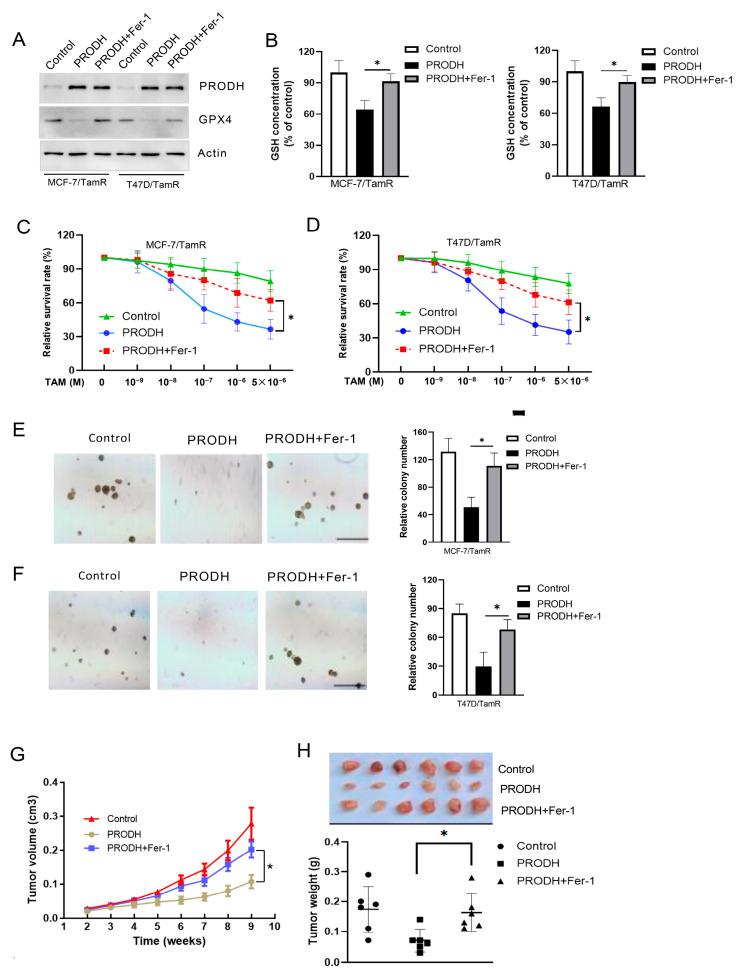
PRODH regulates tamoxifen response through modulation of ferroptosis. (**A**) MCF-7/TamR and T47D/TamR cells were treated with fer-1 and GPX4 expressions were measured by Western blot. (**B**) Cells indicated in A were treated with fer-1, and GSH concentrations were measured. (**C**,**D**) Cells expressing PRODH were treated with TAM and fer-1 for 5 days. MTT assays were performed to detect cell proliferation. (**E**,**F**) Tamoxifen-resistant cells were treated with fer-1 and cell anchor-independent growth was analyzed by soft agar. The scale bar indicates 100 nm. (**G**) The effect of PRODH on tumor growth under tamoxifen treatment. MCF-7/TamR cells expressing PRODH were injected into nude mice. Mice were randomly assigned to 3 groups as indicated and treated with tamoxifen and or fer-1. (**H**) When we reached the endpoint, mice were euthanized and tumors were weighed. * *p* < 0.05.

To investigate whether ferroptosis mediates the role of PRODH in tamoxifen response in breast cancer cells, we treated tamoxifen-resistant cells with a ferroptosis inhibitor fer-1. As shown in Figure 6A, fer-1 treatment inhibited the repressive effect of PRODH on GPX4 expression in MCF-7/TamR and T47D/TamR cells. We also observed that fer-1 treatment diminished the effect of PRODH on ROS and GSH levels in these cells (Figure 6B and Supplementary Figure S1A,B). These findings suggest that fer-1 suppressed ferroptosis induction by PRODH in tamoxifen-resistant cells. Then, our cell anchor-dependent (Figure 6C,D) and independent growth assays (Figure 6E,F) revealed that fer-1 treatment diminished the effect of PRODH on tamoxifen response in MCF-7/TamR and T47D/TamR cells. Finally, we decided to determine whether ferroptosis mediates the role of PRODH in tamoxifen resistance using a nude mouse model. MCF-7/TamR cells stably expressing PRODH were injected into nude mice and treated with tamoxifen and either fer-1 or vehicle. As a result, fer-1 treatment reversed the effect of PRODH on tumor growth under tamoxifen treatment (Figure 6G,H). These findings indicate that ferroptosis mediates the effect of PRODH on tamoxifen resistance in breast cancer cells.

## 4. Discussion

In this study, we have identified the key role of PRODH in tamoxifen response via regulating ferroptosis in breast cancer. We revealed that PRODH expression is reduced in tamoxifen-resistant cells, and PRODH overexpression enhanced the response of resistant cells to tamoxifen. We showed that PRODH knockdown is associated with tamoxifen resistance in tamoxifen-sensitive cells. Finally, we observed that PRODH enhances ferroptosis and regulates tamoxifen resistance through ferroptosis modulation.

Tamoxifen resistance is a major challenge in breast cancer treatments and is indicative of a poor prognosis in breast cancer patients [28]. In rapidly growing tumors, cancer cells require a vast amount of nutrients to support their proliferation. In cancer cells, many metabolic pathways are frequently reprogrammed to meet their energy demands [29]. Recent studies have established a strong association between tamoxifen resistance and metabolism alterations such as glycolysis and glutaminolysis [25,30,31]. PRODH is critical for proline degradation and is a tumor suppressor in many types of cancers [14,16]. This study identified the role of PRODH in tamoxifen response. We observed an increase in the proline content in tamoxifen-resistant cells. In contrast, we observed that PRODH is scarcely detectable in tamoxifen-resistant cells. PRODH overexpression increased the level of proline and enhanced resistant cell response to tamoxifen. Although there is no effect on cell growth in PRODH knockdown MCF-7 and T47D (tamoxifen-sensitive) cells, our study shows that PRODH knockdown conferred tamoxifen resistance in these cells. Another group’s study has shown that *PRODH* mRNA levels positively correlate with the outcomes in breast cancer [23]. These data indicate that PRODH activation is a potential target to reduce tamoxifen resistance. However, a recent study shows that PRODH induced triple-negative breast cancer (TNBC) cell malignant phenotypes and overexpression of PRODH sensitized TNBC cells to clinical therapeutic drugs, including lipodox and Palbociclib [26]. These data suggest that PRODH plays different roles depending on major subtypes of breast cancer.

PRODH catalyzes the oxidation of proline. In this process, electrons can be transferred to the respiratory chain to produce ATP. At the same time, reactive oxygen species (ROS) are sufficiently produced. The ATP production promotes cell survival, while ROS production induces apoptosis [18,19,32,33,34,35]. The mechanism for PRODH-mediated apoptosis is not yet fully understood. Ferroptosis was first proposed in 2012; it is a highly iron-dependent non-apoptotic form of cell death [36,37]. Ferroptosis, a novel iron-induced programmed cell death, is characterized by lipid peroxidation. GPX4 (glutathione peroxidase 4) has been shown to be an important regulator in ferroptosis [38]. Increasing studies suggest that GPX4 is a novel tumor suppressor in many types of cancers. The association between ferroptosis and cancer therapy resistance has been well documented. This implies the potential of triggering ferroptosis for cancer treatments, especially for traditional therapy-resistant tumor therapy [39]. A recent study indicates that proline regulates the level of GSH [40]. This implies a possible association between proline metabolism and ferroptosis. Here, our data suggest that PRODH decreases GPX4 expression and enhances ferroptosis in breast cancer cells. We demonstrate that ferroptosis exhibited a significantly lower level in tamoxifen-resistant cells compared with their parent cells. This indicates that ferroptosis plays an important role in tamoxifen resistance. We also found that PRODH re-expression restored ferroptosis in tamoxifen-resistant cells. Meanwhile, the ferroptosis inhibitor fer-1 diminished the effect of PRODH in tamoxifen resistance. These findings have established the association between proline metabolism and ferroptosis, and they cooperate to regulate tamoxifen response in breast cancer cells. In future studies, we will testify the relationship between PRODH, ferroptosis, and tamoxifen response using mouse models and patient tumor tissues. We will investigate if PRODH expression is an indicator for tamoxifen usage in clinics.

## 5. Conclusions

We identified the role of proline metabolism and PRODH in tamoxifen resistance. PRODH sensitizes breast cancer to tamoxifen through ferroptosis. We also identified the association between ferroptosis and tamoxifen resistance. Future research should investigate the association between GPX4 and PRODH expressions and endocrine therapy outcomes in patients. In patients with low PRODH expression, ferroptosis activator reagents may provide a potential strategy when combined with tamoxifen to treat this type of breast cancer.

## Data Availability

The original contributions presented in the study are included in the article, further inquiries can be directed to the corresponding author.

## Supplementary Materials

The following supporting information can be downloaded at: https://www.mdpi.com/article/10.3390/genes15101316/s1, Figure S1: PRODH regulates ROS levels through modulation of ferroptosis in tamoxifen resistant cells.

## Abbreviations

PRODH, proline dehydrogenase; ER, estrogen receptor; NEAA, non-essential amino acids; POX, proline oxidase; P5C, pyrrolidine-5-carboxylate; GPX4, glutathione peroxidase 4; TamR, tamoxifen resistance; FBS, fetal bovine serum; TAM, 4-Hydroxytamoxifen; ROS, reactive oxygen species; shRNA, short hairpin RNA; GSH, glutathione; TNBC, triple-negative breast cancer; Fer-1, ferrostatin-1.
