# Supplementary material for: PRODH Regulates Tamoxifen Resistance through Ferroptosis in Breast Cancer Cells
Source: Genes (Basel). 2024 Oct 14;15(10):1316. doi: 10.3390/genes15101316 (PMC11507086; doi:10.3390/genes15101316)
Supplement: Supplementary file 1 [file genes-15-01316-s001.zip › genes-3206429-supplementary.pdf]

Supplementary materials

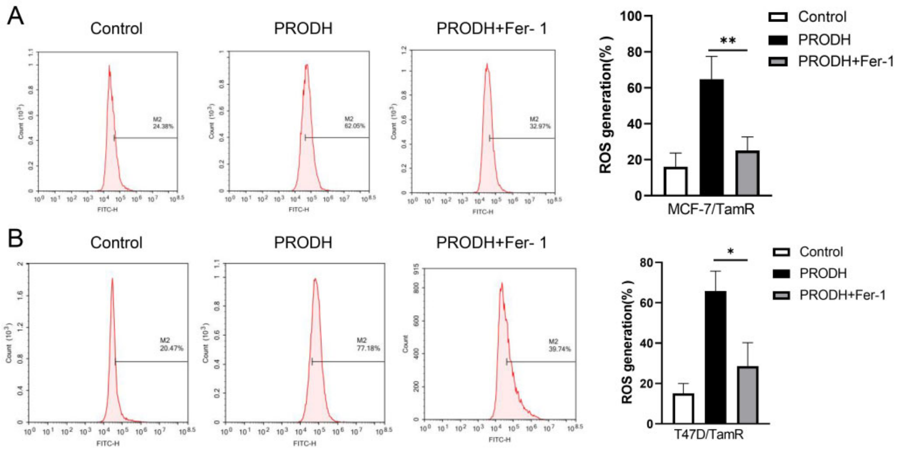

Supplemental Figure S1. PRODH regulates ROS levels through modulation of ferroptosis in tamoxifen resistant cells. **(A, B)** PRODH expressing MCF-7/TamR and T47D/TamR cells were treated with fer-1 and ROS levels were measured.
